# Supporting adherence to oral anticancer agents: clinical practice and clues to improve care provided by physicians, nurse practitioners, nurses and pharmacists

**DOI:** 10.1186/s12885-017-3110-2

**Published:** 2017-02-10

**Authors:** Lonneke Timmers, Christel C. L. M. Boons, Mathieu Verbrugghe, Bart J. F. van den Bemt, Ann Van Hecke, Jacqueline G. Hugtenburg

**Affiliations:** 1 Department of Clinical Pharmacology and Pharmacy, VU University Medical Center, De Boelelaan 1117, 1081 HV Amsterdam, The Netherlands; 2 University Centre of Nursing and Midwifery, Department of Public Health, Ghent University, Ghent, Belgium; 3 Department of Pharmacy, Sint Maartenskliniek, Nijmegen, The Netherlands; 4 Department of Pharmacy, Radboud University Medical Center, Nijmegen, The Netherlands; 5 The EMGO Institute for Health and Care Research, VU University Medical Center, Amsterdam, The Netherlands

**Keywords:** Oral anticancer agents, Medication adherence, Multidisciplinary care, Healthcare providers, Adherence management, Clinical practice, Physician, Nurse, Nurse practitioner, Pharmacist

## Abstract

**Background:**

Healthcare provider (HCP) activities and attitudes towards patients strongly influence medication adherence. The aim of this study was to assess current clinical practices to support patients in adhering to treatment with oral anticancer agents (OACA) and to explore clues to improve the management of medication adherence.

**Methods:**

A cross-sectional, observational study among HCPs in (haemato-)oncology settings in Belgium and the Netherlands was conducted in 2014 using a composite questionnaire. A total of 47 care activities were listed and categorised into eight domains. HCPs were also asked about their perceptions of adherence management on the items: *insight into adherence, patients’ communication, capability to influence, knowledge of consequences* and *insight into causes.* Validated questionnaires were used to assess beliefs about medication (BMQ) and shared decision making (SDM-Q-doc).

**Results:**

In total, 208 HCPs (29% male) participated; 107 from 51 Dutch and 101 from 26 Belgian hospitals. Though a wide range of activities were reported, certain domains concerning medication adherence management received less attention. Activities related to patient knowledge and adverse event management were reported most frequently, whereas activities aimed at patient’s self-efficacy and medication adherence during ongoing use were frequently missed. The care provided differed between professions and by country. Belgian physicians reported more activities than Dutch physicians, whereas Dutch nurses and pharmacists reported more activities than Belgian colleagues. The perceptions of medication adherence management were related to the level of care provided by HCPs. SDM and BMQ outcomes were not related to the care provided.

**Conclusions:**

Enhancing the awareness and perceptions of medication adherence management of HCPs is likely to have a positive effect on care quality. Care can be improved by addressing medication adherence more directly e.g., by questioning patients about (expected) barriers and discussing strategies to overcome them, by asking for missed doses and offering (electronic) reminders to support long-term medication adherence. A multidisciplinary approach is recommended in which the role of the pharmacist could be expanded.

**Electronic supplementary material:**

The online version of this article (doi:10.1186/s12885-017-3110-2) contains supplementary material, which is available to authorized users.

## Background

Due to the availability of rapidly growing numbers of new oral anticancer agents (OACA) directed towards specific tumour cell targets, in (haemato-)oncology medication adherence is becoming an increasingly important issue [1]. Oral administration may improve quality of life by its convenience and ease of use. Provided that efficacy and toxicity are at least similar to the effects of IV treatment, most patients therefore prefer treatment with OACA [2, 3].

Non-adherence to medication is a complex and multidimensional healthcare problem. Adherence is defined as the extent to which a patient follows agreed recommendations for prescribed treatments [4]. Patients may intentionally or unintentionally be non-adherent during different stages of their treatment [5, 6]. Adherence to long-term therapies in chronic diseases is estimated at 50–70% [4, 7]. Regarding adherence to OACA, rates between 16 and 100% have been reported [8].

For the individual patient non-adherence may have serious consequences (e.g., lack of efficacy or increased toxicity) while society may face increased healthcare costs [1]. The minimum level of adherence required to achieve a positive clinical outcome (the so called ‘drug forgiveness’), varies by drug and is often not exactly known. Research on adherence to protein kinase inhibitor treatment in patients with chronic myeloid leukaemia (CML) revealed the existence of a strong relationship between the missing of only a small number of doses per month (5%) and a less favourable clinical outcome [9].

Factors influencing adherence are numerous [10, 11]. The WHO framework elaborately describes the multidimensional phenomenon of medication adherence [4]. It includes five interacting dimensions that influence adherence: social and economic factors, condition-related factors, therapy-related factors, patient-related factors, and healthcare provider (HCP) and system-related factors. Few studies have been published on HCP-related factors influencing medication adherence in (haemato-)oncology. In patients with breast cancer on chronic endocrine therapy, a poor physician’s explanation of treatment effects was related to non-adherence [12]. In addition, patients with CML on long-term imatinib treatment reported that positive feedback from physicians reinforced the belief that ‘occasional’ non-adherence would not affect efficacy [13]. More generally, it appeared that HCPs’ beliefs about OACA affected their behaviour and care attitude which in turn influenced the adherence behaviour of patients [13].

HCPs’ beliefs about OACA and their use, HCPs’ perceptions of OACA adherence management and in the case of physicians their perceptions of shared decision making, were explored in the first part of this study which was published separately [14]. Most HCPs considered themselves to have adequate knowledge of the causes and consequences of non-adherence and felt able to influence the medication adherence of their patients. However, several HCPs, nurses and pharmacists in particular, appeared to have no information on the actual adherence of their patients, nor did they thought that their patients were willing to discuss adherence with them. Unfortunately, it remained unclear to what extent these findings were related to the care that was provided.

In supporting patients to adhere to their medication HCPs must create several preconditions for adherent behaviour [15, 16]. In this respect, patients need to be aware of the existence and consequences of non-adherence and need to be convinced that they have the capacity to manage their treatment themselves (self-efficacy). In addition, they must have been given clear instructions on how to use the prescribed medication and must be able to correctly use the medication. Knowledge of their disease and treatment is therefore also needed as well as social support, adverse event management instructions and removal of all possible barriers to the optimal use of OACA.

The aim of this study was to explore current clinical practices of supporting adherence to treatment with OACA in Belgium and the Netherlands and to find clues for improvement of care. Furthermore, the relationship between HCPs’ beliefs and the supportive care HCPs provide have been explored.

## Methods

### Study design

Using a cross-sectional observational design, the present study was conducted in the period April - October 2014 in the Netherlands and Belgium. HCPs with the profession of medical oncologist, haematologist, nurse practitioner (NP), nurse or pharmacist, and providing patient care in a (haemato-)oncology setting in the Netherlands or Dutch-speaking part of Belgium, were asked to fill out a questionnaire.

### Data collection procedure

HCPs were invited by their professional associations to fill out an electronic questionnaire available in the form of a secure internet-link. The professional associations that spread the link to their members were the NVMO, HOVON, NVALT, V&VN, NVPF in the Netherlands and the VZA, VVRO, BSMO, BHS in Belgium. The link was made available either by e-mail and/or publication in an electronic newsletter. A reminder was sent to stimulate response. Additional recruitment took place by distributing the internet-link within the authors’ network, and by handing out a paper version of the questionnaire at a scientific meeting on adherence to OACA treatment, held on the 13^th^ of October 2014 in Brussels. The participants completed the questionnaire anonymously.

### Questionnaire

A composite questionnaire was used, starting with personal characteristics of the respondents i.e., profession, gender, number of years employed, hospital and specialization. The questionnaire consisted of four parts: 1) Perceptions of adherence management, 2) Shared decision making (SDM), 3) Beliefs about OACA, and 4) Care usually provided (usual care (UC)) in supporting adherence to treatment with OACA. As respondents were not able to give multiple answers for different patient groups, they were asked to complete the questionnaire in relationship to their main patient group (the patient group they treat most frequently). The questionnaire was pilot-tested by nine HCPs (i.e., a medical oncologist, a haematologist, three nurse practitioners, three pharmacists, and a general practitioner) in Belgium and the Netherlands. In individual interviews it was explored whether the items were understood as intended. The pilot-HCPs were also asked about items of care activities to support medication adherence and to add items if anything was missing. After processing the comments, the final version of the questionnaire was defined.

1. Perceptions of medication adherence management
  To assess HCPs’ perceptions of medication adherence management, five questions were formulated: (1) *Insight into adherence:* I know the level of adherence of all my patients; (2) *Patients’ communication:* I think that patients discuss non-adherence with me, (3) *Capability to influence:* I am able to influence adherence behaviour of my patients, (4) *Knowledge of consequences:* I have sufficient knowledge of the consequences of non-adherence, and (5) *Insight into causes:* I have sufficient knowledge of the causes of non-adherence to discuss this with patients. Answers were given on a 5-point Likert scale (where 1 = strongly disagree, 2 = disagree, 3 = uncertain, 4 = agree and 5 = strongly agree). The answers ‘agree’ and ‘strongly agree’ were dichotomized into ‘yes’ [1] and the remaining answers into ‘no or uncertain’ (0). A sum score of the Perceptions of Adherence Management Questions (PAMQs sum score), ranging from zero to five, was calculated by summing the five dichotomized items.
2. Shared decision making
  The validated Shared Decision Making Questionnaire – physician version (SDM-Q-Doc), in the authorized Dutch translation, was used [17]. The SDM-Q-Doc consists of nine items that are rated on a 6-point Likert scale (‘completely disagree’ to ‘completely agree’, scored with 0 to 5). A sum score was made (range 0 to 45), and linear transformed into a scale from zero to 100 [15]. A higher score indicates a higher level of acceptance towards shared-decision making.
3. Beliefs about OACA and their use
  The validated Beliefs about Medicines Questionnaire (BMQ-Specific) [18, 19] was incorporated to assess the beliefs about the necessity of the medication to control the disease and the concerns about the potential negative impact of the medication. The BMQ-Specific consists of five items for the subscales ‘Necessity’, and ‘Concerns’. Both are scored on a 5-point Likert scale (‘strongly disagree’ to ‘strongly agree’, scored from 1 to 5) resulting in a score for the subscales ranging from 5 to 25. BMQ-Specific was adapted for use in HCPs by Lesuis et al. (Sint Maartenskliniek, Nijmegen, the Netherlands) and was translated into Dutch according to the inverse translation method [20] by CB and LT. The Dutch HCP version was authorised by the original first author R. Horne [18]. HCPs were categorized into four attitudinal groups: accepting (high necessity, low concerns), ambivalent (high necessity, high concerns), indifferent (low necessity, low concerns) and sceptical (low necessity, high concerns) with the scale midpoint of 15 or above used as a cut-off to define low and high beliefs [21].
4. Care usually provided in supporting adherence to treatment with OACA
  To assess the care provided in supporting adherence to treatment with OACA, a list of care activities was prepared. Point of departure of the list was the Quality of Standard Care questionnaire as used by the Bruin et al. [15, 16] to assess usual care in supporting patients to adhere to anti-retroviral therapy. The list was adapted to cancer care by the research team consisting of three pharmacists, a nurse, a psychologist and a health scientist, with expertise in the field of medication adherence in medical oncology and haematology. A total of 47 care activities were listed. Items were divided into three parts: activities carried out at the initiation of therapy, activities carried out during follow-up appointments, and activities which were not connected to specific time-points. For each item, HCPs were asked to indicate whether they had provided that particular care (activity) during the last six months to the majority of their patients. When the answer was positive, this activity was calculated with one point in the sum score. The minimum score is zero (when none of the listed care activities usually performed), the maximum score is 47 (when all 47 care activities are usually performed).

The listed items were categorised into eight domains: *Knowledge, Awareness, Self-efficacy, Intention Formation, Implementation, Social Support, Adverse Events Management* and *Facilitation*. Each member of the research group independently categorised the 47 care activities into one of the eight domains. The categorization of the items was discussed within the research group in two rounds until consensus was reached. Table 1 gives an overview of the domains, its definitions, and typically used techniques within the domain. The categorization was not made with the intention to develop a questionnaire that assesses eight domains, but was done to organize all the activities.

**Table 1:** Domains of Usual Care activities in supporting adherence to OACA

Domain	Definition	Typically used technique(s)
Knowledge	Usual care activities focussing on the knowledge of patients about their diseases and the medicines used for treatment, excluding knowledge related to adverse events	- providing information - increase patient understanding
Awareness	Usual care activities aimed to increase the awareness of patients with respect to non-adherence to treatment and consequences of non-adherence	- risk communication - giving feedback on patients’ behaviour
Self-efficacy	Usual care activities that focus on self-efficacy; a patient’s belief in her/his ability in succeeding to adhere to treatment	- the planning of coping responses like discussing barriers and finding ways to overcome them
Intention Formation	Usual care activities which focus on fostering the intention to adhere by planning how and when to take the medication	- tailoring the medication schedule
Implementation	Usual care activities which focus on the effective implementation of the intended use of medication	- stimulating the use of cues
Social Support	Usual care activities that provide patients with professional social support with respect to the correct use of their medication	- giving social support
Adverse Events Management	Usual care activities which focus on patients’ management of adverse events	- providing information about adverse events - facilitating coping with adverse events
Facilitation	Usual care activities which facilitate a correct use of medication and which are not categorized in one of the other domains	- reducing environmental barriers

### Statistics

Respondent descriptive data were analysed as frequencies (percentages) for categorical variables and as the median and interquartile range (IQR) for continuous data. The usual care sum scores of HCPs in the Netherlands and in Belgium were compared for all professions by means of the non-parametric Mann Whitney test for nurse practitioners and the *T*-test for all other professions (with normally distributed scores). Though the items listed Associations between respondent characteristics and care activities were assessed in univariate linear regression analyses, with the usual care sum score as the dependent variable. A multivariate linear regression was performed using all HCPs’ characteristics with p < 0.25 in the univariate analyses. A backward elimination procedure was used where at each step the predictor with highest *p*-value was dropped from the model until only significant predictors remained. For all analyses, a two-tailed significance level of 0.05 was used. *P*-values below this level were considered statistically significant. Statistical analysis was performed with SPSS 22.0 for Windows (IBM Corp, Armonk, NY, USA).

## Results

### Respondent characteristics

A total of 208 HCP (29% male) participated, of whom 107 were affiliated to 51 of 95 (54%) hospitals in the Netherlands and 101 were affiliated to 26 of 59 (44%) hospitals in Belgium. Of the participants 31.8% was physician (15.9% medical oncologist, 15.9% haematologist), 28.8% nurse, 16.8% nurse practitioner and 22.6% pharmacist. HCP characteristics and their scores on the PAMQs, SDM-Q-doc, and BMQ are shown in Table 2.

**Table 2:** Characteristics of health care providers *N* = 208

	All	NL	Be
	*N* = 208	*N* = 107	*N* = 101
Gender (%)			
Male	29.3	33.6	24.8
Female	70.7	66.4	75.2
Profession (%)			
Medical oncologist	15.9	10.3	21.8
Haematologist	15.9	26.2	5.0
Nurse practitioner	16.8	17.8	15.8
Nurse	28.8	21.5	36.6
Pharmacist	22.6	24.3	20.8
Work experience (yr)			
Median	16	16	17
Range	1–46	2–46	1–40
Type of hospital (%)			
Academic	29.6	22.4	37.4
Non-academic	70.4	77.6	62.6
Number of hospitals	87	51	36
Specialisation (%)			
Haematology	30.6	38.0	22.6
Oncology	69.4	62.0	77.4
Adherence (PAMQs) (%)			
Insight into adherence	41.8	43.9	39.6
Patients’ communication	43.8	45.8	41.6
Capability to influence	82.2	86.0	78.2
Knowledge of consequences	78.8	75.7	82.2
Insight into causes	68.3	69.2	67.3
PAMQs sum score (0–5)			
Median	3.0	3.0	3.0
IQR	2.0–4.0	2.0–4.0	2.0–4.0
SDM^a^-score (0–100)	82.2	84.4	80.0
BMQ-Specific (mean ± sd)			
Necessity	18.3 ± 3.0	17.8 ± 2.9	18.7 ± 3.0
Concerns	13.4 ± 2.6	12.9 ± 2.6	13.9 ± 2.5
N-C differential	4.9 ± 3.7	4.9 ± 3.8	4.8 ± 3.7
BMQ-group (%)			
Accepting	58.3	61.3	55.0
Ambivalent	31.1	24.5	38.0
Indifferent	8.3	11.3	5.0
Sceptical	2.4	2.8	5.0

*Abbreviations: NL* the Netherlands, *Be* Belgium, *yr* year, *PAMQs, HCP’s* Perceptions of Adherence Management Questions, *IQR* interquartile range, *SDM-score* sum score of the Shared Decision Making-doc-Questionnaire, *BMQ* Beliefs about Medicines Questionnaire

^a^SDM assessed only for physicians

### Usual care provided in supporting adherence to OACA treatment

Table 3 depicts for each of the 47 care activities the percentage of physicians, nurse practitioners, nurses and pharmacists who reported to perform this care activity in the last six months in the majority of their patients. The Cronbach’s alpha for the domains of care activities are: *Knowledge*: 0.836*, Awareness*: 0.693*, Self-efficacy*: 0.886, *Intention Formation*: 0.754, *Implementation*: 0.548, *Social Support*: 0.519, *Adverse Events Management*: 0.909 and *Facilitation*: 0.791. In the Additional file 1: Table S1 they are presented by profession as well. The median score and interquartile range per domain are shown in Table 4. The median usual care sum score (range 0–47) was 24.0, 30.0, 24.5 and 11.0 for physicians, nurse practitioners, nurses and pharmacists, respectively. The median scores as percentage of the maximum score for physicians, NPs, nurses and pharmacists, respectively, were: *Knowledge*: 86, 100, 71 and 29%; *Awareness*: 75, 75, 63 and 0%; *Self-efficacy*: 60, 80, 50 and 0%; *Intention Formation*: 67, 100, 83 and 50%; *Implementation*: 25, 50, 25 and 0%; *Social Support*: 67, 67, 67 and 0%; *Adverse Events Management*: 100, 100, 100 and 29%; *Facilitation*: 64, 73, 55 and 27%.

**Table 3:** Usual Care activities in supporting adherence to OACA *N* = 208

		physician		NP		nurse		pharmacist	
		NL 39^a^	Be 27^a^	NL 19^a^	Be 16^a^	NL 23^a^	Be 37^a^	NL 26^a^	Be 21^a^
		%	%	%	%	%	%	%	%
Knowledge	PoT								
Provide information on the disease	S	100.0	100.0	100.0	100.0	82.6	70.3	23.1	9.5
Provide information on the expected effect(s) of the drug	S	100.0	100.0	100.0	75.0	86.4	59.5	38.5	19.0
Discuss the action of the drug	S	92.3	100.0	100.0	75.0	81.8	48.6	57.5	23.8
Hand out brochures or written information about the disease and/or medication used for treatment	S	62.2	74.1	84.4	87.5	87.6	54.1	84.6	28.6
Discuss when the first effect of the medication can be expected	S	100.0	100.0	84.4	56.3	72.7	37.8	26.9	14.3
Monitor and/or discuss possible interactions with other medicines or foods	S	75.7	96.3	100.0	87.5	73.9	62.2	96.2	33.3
Discuss (changes in) sexuality	G	35.1	28.0	82.4	50.0	47.4	35.1	0.0	0.0
Awareness									
Discuss the importance of treatment adherence	S	84.2	88.5	82.4	93.8	85.0	78.4	64.0	28.6
Discuss the consequences of non-adherence (to treatment)	S	64.9	80.8	70.6	68.8	65.6	56.8	32.0	23.8
Ask the patient if he/she has missed one or more doses	F	68.4	60.0	94.1	68.8	78.9	59.5	12.0	19.0
Discuss the use and results of the Medication Event Monitoring System (MEMS)	F	2.8	8.0	6.3	6.3	5.3	5.4	4.0	0.0
Self-efficacy									
Encourage patients to timely plan the intake of medicines during holidays and weekends	S	52.8	53.8	58.8	56.3	47.4	40.5	24.0	19.0
Discuss potential barriers regarding treatment adherence	S	51.4	61.5	68.8	56.3	65.0	45.9	28.0	23.8
Discuss possible ways to overcome potential barriers regarding treatment adherence	S	51.4	65.4	76.5	62.5	65.0	40.5	32.0	23.8
Inquire after barriers regarding treatment adherence	F	55.3	64.0	82.4	68.8	57.9	45.9	20.0	14.3
Discuss ways to overcome potential barriers regarding treatment adherence	F	51.4	64.0	76.5	62.5	57.9	35.1	20.0	14.3
Intention Formation									
Discuss the scheduled duration of medication treatment	S	100.0	100.0	100.0	81.3	77.3	45.9	46.2	14.3
Explain how often the medicine should be taken. If necessary, explain the treatment schedule	S	84.6	100.0	100.0	93.8	91.3	89.2	96.2	52.4
Discuss the intake of the medicines relative to that of meals and why	S	60.0	88.9	100.0	93.8	91.3	86.5	92.3	47.6
Discuss what to do if there is vomiting shortly after ingestion of the medicine	S	41.7	85.2	88.9	93.8	81.8	73.0	38.5	28.6
Explain what to do if a dose is missed	S	51.4	76.9	94.1	100.0	94.7	64.9	48.0	38.1
Development of an individual written medication schedule	S	17.1	42.3	75.0	87.5	52.6	48.6	32.0	23.8
Implementation									
Identify daily routines and encourage patients to align the taking of medicines with their routines	S	45.9	69.2	100.0	81.3	90.0	62.2	28.0	19.0
Encourage patients to use a seven day pillbox	S	13.5	23.1	50.0	50.0	21.1	43.2	16.0	19.0
Encourage patients to use the Medication Event Monitoring System (MEMS)	S	0.0	11.5	12.5	6.3	0.0	5.4	4.0	4.8
Encourage patients to use alarm devices for properly timing their medication intake	S	5.6	19.2	58.8	37.5	21.1	29.7	12.5	14.3
Social Support									
Involve partner and/or relatives in the treatment	S	86.8	84.6	87.5	87.5	85.0	81.1	32.0	23.8
Encourage patients to organize social support	G	55.6	40.0	58.8	50.0	60.0	48.6	4.0	0.0
Refer a patient to a patients’ association	G	73.7	24.0	76.5	43.8	47.4	21.6	4.0	0.0
Adverse Events Management									
Discuss the common adverse events of the drug	S	94.9	100.0	100.0	87.5	87.0	83.8	61.5	28.6
Discuss options to mitigate the impact of adverse events (at start of treatment)	S	70.3	96.3	97.1	93.8	85.0	83.8	48.9	33.3
Discuss the possibility of dose adjustment if adverse events occur	S	86.8	96.3	77.8	68.8	77.3	62.2	26.9	28.6
Inquire after (perceived) adverse events of treatment	F	100.0	100.0	100.0	100.0	89.5	83.8	72.0	23.8
Inquire after the severity of the adverse events	F	100.0	100.0	100.0	93.8	89.5	86.5	56.0	23.8
Discuss options to mitigate the impact of adverse events (during treatment)	F	89.5	96.0	100.0	87.5	94.7	73.0	52.0	28.6
Give the patient a telephone number and tell who to contact in the case of adverse events	G	82.9	88.0	100.0	93.8	94.7	73.0	24.0	19.0
Facilitation									
Explain how and where the product is available	S	81.6	85.2	94.4	87.5	90.5	59.5	76.9	42.9
Discuss drug storage recommendations	S	16.7	55.6	64.7	81.3	70.0	62.2	88.5	81.0
Give feedback about treatment efficacy	F	100.0	100.0	82.4	56.3	73.7	35.1	16.0	9.5
Inquire after positive effects of treatment	F	100.0	92.0	76.5	86.3	89.5	59.5	28.0	19.0
Ensure the timely transfer of medication information to other health care providers	G	86.5	72.0	62.5	56.3	60.0	29.7	100.0	23.8
Call the patient after the start of treatment to ask about experiences	G	11.8	4.0	64.7	37.5	68.4	18.9	8.0	0.0
Give the patient a telephone number and tell who to contact in case of problems with treatment adherence	G	69.4	64.0	82.4	93.8	78.9	54.1	28.0	19.0
Inform the patient about 24 hour availability of assistance	G	91.7	76.0	100.0	62.5	90.0	64.9	28.0	23.8
Intensify the number of follow-up visits if patients have problems with treatment adherence	G	45.9	28.0	58.8	43.8	42.1	18.9	8.0	9.5
Refer patients to another health care provider for (co-) treatment (e.g., in the case of adverse events)	G	57.9	64.0	70.6	56.3	47.4	40.5	40.0	14.3
Refer to another health care provider in case of (suspected) psychosocial problems	G	75.7	80.0	88.2	75.0	78.9	75.7	0.0	14.3

*Abbreviations: OACA* oral anticancer agents, *NL* the Netherlands, *Be* Belgium, *PoT* point of time of the activity, *S* at start of treatment, *F* during follow-up visits, *G* general activity which is not attached to a time-point, *NP* nurse practitioner

^a^missings excluded from analyses

**Table 4:** Usual Care in supporting adherence to OACA: median scores per domain

		Awareness	Self-efficacy	Intention Formation	Implemen- tation	Social Support	Adverse Events Management	Facilitation	UC sum score
Range:	0–7	0–4	0–5	0–6	0–4	0–3	0–7	0–11	0–47
Physicians									
Median	6.0	3.0	3.0	4.0	1.0	2.0	7.0	7.0	24.0
IQR	5–6	2–3	1–5	3–5	0–1	1.5–2	6–7	6–8	19.3–28.8
% median score	86%	75%	60%	67%	25%	67%	100%	64%	51%
NPs									
Median	7.0	3.0	4.0	6.0	2.0	2.0	7.0	8.0	30.0
IQR	6–7	2–3	1.3–5	6–6	1–3	2–3	6–7	7–10	25.5–34.0
% median score	100%	75%	80%	100%	50%	67%	100%	73%	64%
Nurses									
Median	5.0	2.5	2.5	5.0	1.0	2.0	7.0	6.0	24.5
IQR	3–6	1–3	0–5	3–6	1–2	1–3	5.3–7	4–9	15.8–31.0
% median score	71%	63%	50%	83%	25%	67%	100%	55%	52%
Pharmacists									
Median	2.0	0	0	3.0	0	0	2.0	3.0	11.0
IQR	0–3.3	0–2	0–2	1–4	0–1	0–1	0–6	1–6	3.5–17.0
% median score	29%	0%	0%	50%	0%	0%	29%	27%	23%

*Abbreviations: UC* usual care, *IQR* interquartile range, *NPs* nurse practitioners

### Belgium versus the Netherlands

Table 5 shows the mean usual care sum scores of the different professions for the Netherlands and Belgium separately. Belgian physicians had a higher UC sum score compared to their Dutch colleagues (31.0 vs. 22.7) (*p* = 0.043). Dutch nurses and pharmacists had a higher UC sum score than their Belgian colleagues (35.0 vs. 28.0 and 18.5 vs. 3.0, respectively *p* < 0.001 and *p* =0.026).

**Table 5:** Usual Care in the Netherlands versus Belgium

	NL		Be		NL vs. Be
	N	UC-sum	N	UC-sum	*p*
Physician	27	22.7	25	31.0	0.043*
NP	13	38.0	16	36.5	0.263
Nurse	17	35.0	37	28.0	<0.001*
Pharmacist	24	18.5	21	3.0	0.026*

*Abbreviations: vs* versus, *NL* the Netherlands, *Be* Belgium, *UC-sum* mean sum score of usual care activities (0-47); *NP* nurse practitioner

*significant (*p* < 0.05)

### Associations with usual care provided in supporting adherence to treatment with OACA

Univariate and multivariate associations with the usual care sum score are presented in Table 6. Compared with physicians, the usual care sum score of nurse practitioners was higher (beta 4.2; 95%CI [0.4, 8.0], *p* = 0.031) and the usual care sum score of pharmacists was lower (beta -12.7; 95%CI [-16.0, -9.3], *p* < 0.001). Perceptions of adherence management were related to the care provided. A more positive score on the perceptions questions (higher PAMQs sum score) was significantly related to a higher usual care sum score (beta 3.2; 95%CI [2.3, 4.2], *p* < 0.001). Higher scores on the PAMQ *Insight into adherence*, *Patients’ communication*, *Capability to influence* and *Insight into causes* were significantly related with a higher usual care sum score (*p* < 0.02). In the multivariate linear regression analyses the following HCPs’ characteristics were significantly associated with the usual care sum score: Profession (*p* < 0.001), Country (the Netherlands as reference) (beta -3.5; 95%CI[-5.8, - 1.2], *p =* 0.003), gender (male as reference) (beta 2.8; 95%CI [0.1–5.4], *p* = 0.042) and PAMQs sum score (beta 2.3; 95%CI [1.4, 3.1], *p* < 0.001). The beliefs about OACA and the physicians’ perceptions about SDM were not associated with the usual care sum score.

**Table 6:** Associations with Usual Care sum score *N* = 180

	univariabel			multivariabel		
	beta	95% CI	*p*-value	beta	95% CI	*p*-value
Gender (male)	1.97	[–1.35, 5.28]	0.244	2.75	[0.096, 5.40]	0.042
Profession			<0.001*			<0.001*
Physician as reference:						
Nurse Practitioner	4.19	[0.38, 8.01]	0.031*	3,2	[–0.43, 6.83]	0.084
Nurse	–1.60	[–4.80, 1.59]	0.324	–0,54	[–3,64, 2.55]	0.729
Pharmacist	–12.69	[–16.04,–9.34]	<0.001*	–10,9	[–14.2,–7.72]	< 0.001
Work experience (yr)	–0.03	[–0.17, 0.11]	0.673			
Type of hospital (academic)	–3.20	[–6.45, 0.06]	0.054			
Specialisation (oncology)	–2.36	[–5.80, 1.09]	0.178			
Country (the Netherlands)	–2.51	[5.50, 0.48]	0.100	–3,51	[–5.81,–1.21]	0.003*
Adherence (PAMQs)						
Insight in adherence	6.80	[3.93, 9.68]	<0.001*			
Patients’ communication	9.32	[6.62, 12.01]	<0.001*			
Capability to influence	5.78	[2.08, 9.47]	0.002*			
Knowledge of consequences	3.60	[–0.04, 7.24]	0.053			
Insight in causes	3.70	[0.59, 6.81]	0.020*			
PAMQs sum score	3.23	[2.26, 4.19]	<0.001*	2,26	[1.41, 3.11]	<0.001*
SDM score^a^	–0.00	[–0.09, 0.09]	0.973			
BMQ-Specific						
Necessity	–0.15	[–0.65, 0.36]	0.565			
Concerns	–0.074	[–6.94, 0.55]	0.814			
N-C differential	–0.07	[–0.48, 0.35]	0.753			
BMQ-group			0.972			
Accepting as reference:						
Ambivalent	–3.34	[–3.72, 3.05]	0.844			
Indifferent	0.32	[–5.45, 6.08]	0.866			
Sceptical	–2.22	[–12.55, 8.11]	0.680			

*Abbreviations*: *OR* odds ration; 95%CI, 95% confidence interval, *yr* year, *PAMQs* Perceptions of Adherence, Management Questions, *SDM-score* sum score of the Shared Decision Making-doc-Questionnaire, *BMQ* Beliefs about Medicines Questionnaire, *N-C* Necessity-Concerns

^a^SDM only assessed for physicians

* = significant

## Discussion

The present study shows that HCPs considered themselves to actively support their patients in adhering to treatment with OACA by using a wide range of activities. The 47 listed care activities were all, to a greater or lesser extent, performed in clinical practice in the Netherlands and Belgium. However, in certain areas activities were carried out only to a limited extent.

The domain *Knowledge* consists of care activities that are mainly performed at the start of treatment. Providing information is required since patients need to understand the usefulness of a particular drug in order to consent to treatment. Patient education is often used in interventions to enhance medication adherence [22, 23]. However, to achieve awareness of the importance of adhering to OACA treatment the impact of non-adherence should be made clear. Most HCPs reported to discuss both the importance of adherence and the consequences of non-adherence. This is in line with their scores on the PAMQs where the majority of HCPs stated to have adequate knowledge about the consequences of non-adherence [14]. Care to maintain awareness, as reflected by the item ‘ask if a dose is missed’, is provided less frequently, particularly in Belgium. A study on nursing practices for patients on OACA treatment in Japan also found that nurses were less likely to ask patients with refills adherence-related questions [24]. Only a minority of HCPs performed usual care activities within the domain *Self-efficacy*. It is known that self-efficacy is an important factor influencing medication adherence and adequate self-management. It is addressed in theoretical behavioural frameworks [25] as well as in medication adherence oncology research [26, 27]. To raise self-efficacy (expected) barriers to optimal adherence must be identified and strategies to overcome these obstacles should be discussed. This requires HCPs to directly focus on medication adherence. Clear instructions are needed to finish the *Intention Formation*. Instructing patients about the regular intake is reported by almost all HCPs, but information to handle specific situations, for example what to do in case of a missed dose or in case of vomiting shortly after ingestion, is provided less frequently. This item clearly needs more attention. Activities classified in the domain *Implementation* also received relatively poor attention. Care activities within this domain focus on cues that are relevant to prevent unintentional non-adherence. Since adherence decreases by treatment duration [10, 28], care activities aimed at the continuation of a correct use are particularly relevant in long term treatment. In view of the current progress in selecting patients that will respond on OACA treatment, the number of patients on long-term OACA treatment is likely to increase considerably. Thus, there is a growing necessity to support on-going optimal use of OACA. Patients with support from their social environment are generally more adherent than those with insufficient support [29, 30]. Any opportunity to strengthen social support should not be missed. Adverse events generally have the full attention of HCPs. Most physicians, nurse practitioners and nurses performed all care activities within this domain. This finding is not surprising, as in oncology (serious) adverse events frequently occur. Adverse events may substantially impinge on the quality of life [29] and are related to non-adherence and early discontinuation of OACA use [10, 27, 30]. All physicians reported to inquire after experienced adverse events and their severity. In the case of more severe adverse events physicians must adjust OACA dosing regimens in an individual manner. For some OACA this can be accomplished without compromising efficacy [31, 32]. Obtaining information on the occurrence of adverse events and how they were experienced, as well as attempts to alleviate their symptoms are therefore common activities in oncology care. It is well known that unpleasant experiences regarding adverse events are associated with a lower level of medication adherence and higher levels of treatment discontinuation [4, 10]. The last domain, *Facilitation*, includes a variety of care activities. With respect to certain items there are striking differences between Belgian and Dutch HCPs. All Dutch pharmacists reported to ensure the timely transfer of medication information to other HCPs, whereas this is usual care for only a quarter of their Belgian colleagues. This suggests that there is a difference in the national organisation of information exchange between HCPs. It is interesting to note that in both countries the majority of HCPs usually does not intensify follow-up visits in the case that patients have problems with medication adherence.

Not all care activities can be and should be provided to all patients. Care should be tailored to the each patients’ situation and needs. On the other hand, all care domains appear to be relevant in maintaining medication adherence. We therefore recommend to cover all domains. Our list with care activities classified in domains can be used as a starting point to reflect on the level of care in one’s own clinical practice. Furthermore, in intervention studies researchers should be aware of the need to accurately describe both the standard or usual care. In clinical trials too often the control arm has been poorly defined [33, 34], resulting in uncertainty about the effects of the intervention studied [15, 33]. The differences in usual care activities between both studied countries reinforce this need.

The care provided usually to support medication adherence reported in this study differed among professions and country. Whereas in Belgium physicians performed more care activities to support adherence to OACA treatment, in the Netherlands a higher percentage of nurses and pharmacists reported to perform these activities. In line with their specialization, training in education, focus on self-management support and time spent on patient-contact, both in Belgium and in the Netherlands nurse practitioners performed the widest range of care activities. The impact of nurse practitioners on the quality of care in oncology has been shown previously [34, 35]. On the other hand, there was a large difference in care provided by pharmacists in both countries, with Dutch pharmacists performing considerably more activities than their Belgian colleagues. An explanation might be that in Belgium OACA are dispensed by hospital pharmacists, whereas in the Netherlands OACA are dispensed by specialized pharmacies in the outpatient clinics which resemble community pharmacies and are staffed by pharmacists who are trained in patient contact. In addition, Dutch pharmacists generally have access to a patients’ list of (co-)medication due to integrated electronic data services. Nevertheless, in both countries pharmacists only play a limited role in supporting adherence to OACA as compared to other HCPs. Since they are medicine experts and are well experienced in supporting medication adherence in patients with chronic diseases, greater involvement of pharmacists in the multidisciplinary teams may improve adherence care in (haemato-)oncology [36].

Successful care with regard to medication adherence, should not be dependent on individual HCPs but supported by a proper organization of care. Recent studies in other countries on current practices to support patients treated with OACA have revealed considerable variation in the extent and quality of the care provided [24, 37, 38]. A large survey among nurses in the US showed that in about half of practices policies and procedures to support patients were lacking and that interdisciplinary communication was inadequate [37]. A study among Spanish oncology pharmacists also demonstrated that adherence practices for oral OACA treatment were only implemented in about half of hospitals [38]. A nurse-based survey in Japan indicated that adherence-related practices varied and were associated with nurse’s background, type of treatment and healthcare system-related factors [24]. In line with the results of the present study, medication adherence management in patients treated with OACA as part of the care that is usually provided clearly shows opportunities for improvement.

For all HCPs participating in the present study, there was a strong relationship between the perceptions of medication adherence management and the number of care activities performed. Although the majority of HCPs stated to have adequate knowledge of medication adherence management [14], the association suggests that promoting HCPs’ awareness and increasing their knowledge about adherence management will improve the usual care that is provided to support patients in adhering to OACA treatment.

There are strengths and limitations to discuss. The present study provides an extensive survey of care activities performed by a variety of HCPs including physicians, nurse practitioners, nurses and pharmacists aimed to support adherence to OACA treatment. The list of 47 items was literature based and completed with input from medical oncologists, haematologists, nurse practitioners, nurses, pharmacists and researchers experienced in performing care activities related to promoting adherence to OACA treatment from two countries. A limitation to address is that these care activities were reported for patients using OACA for all types of cancer. Patients using long-term medication need to be supported in a different manner than patients with shorter life expectancies. Another limitation is that the response rate could not be calculated. Information on the number of HCPs reached with the postings and the number of the reached HCPs involved in the care for patients using OACA was not available. However, the respondents were employed in no less than 87 hospitals in the Netherlands and Belgium. Another limitation is the potential selection bias as the result of the methods applied. The questionnaire might have been filled out mainly by HCPs with awareness of the importance of medication adherence and/or those actively involved in the management of medication adherence. Furthermore, answers may be overstated by the tendency to give socially desirable answers. It is therefore not unlikely that in daily practice the medication adherence care activities are less extensively performed than reported. Finally, it would also be interesting to study these care activities from the patients’ perspective.

## Conclusions

Although HCPs reported to perform a wide range of care activities, certain domains related to the management of medication adherence in patients treated with OACA were given less attention. Activities related to patient knowledge and adverse event management were reported most frequently but activities aimed to support self-efficacy and maintain adherence during ongoing use were frequently missed. HCPs should improve care by addressing adherence directly e.g., by questioning patients’ (expected) barriers and discussing strategies to overcome them, by asking after missed doses and offering (electronic) reminders to support long-term medication adherence. Enhancing HCP’s perceptions of management of adherence is likely to have a positive effect on the quality of the care they provide to their patients. A multidisciplinary approach is recommended in which the role of the pharmacist could be expanded.

## Additional file

Additional file 1: Table S1. Internal validity of non-validated questionnaires. This table describes the internal validity (Cronbach’s alpha) of the 8 Usual Care Domains (Knowledge, Awareness, Social Influence, Self-efficacy, Intention Formation, Implementation, Adverse Events Management, Facilitation), the Usual care sum score, the PAMQs sum score, the SDM score and the BMQ Necessity and BMQ Concerns subscales for all professions (physicians, nurse practitioners, nurses and pharmacists) as well as for all healthcare providers together. (DOCX 18 kb)

## Abbreviations

BHS: Belgian hematology society
BMQ: Beliefs about medicines questionnaire
BSMO: Belgian society of medical oncology
CI: Confidence interval
CML: Chronic myeloid leukemia
HCP: Healthcare provider
IQR: Interquartile range
NP: Nurse practitioner
NVALT: Nederlandse Vereniging van Artsen voor Longziekten en Tuberculose
NVMO: Nederlandse Vereniging voor Medische Oncologie
NVPF: Nederlandse Vereniging voor Poliklinisch Farmacie
OACA: Oral anticancer agents
PAMQ: Perceptions of adherence management questionnaire
Q: Questionnaire
SDM: Shared decision making
UC: Usual care
V&VN: Verpleegkundigen & Verzorgenden Nederland
VVRO: Vereniging voor Verpleegkundigen Radiotherapie en Oncologie
VZA: Vlaamse Vereniging van Ziekenhuisapothekers
WHO: World Health Organisation
