# Supplementary material for: Supporting adherence to oral anticancer agents: clinical practice and clues to improve care provided by physicians, nurse practitioners, nurses and pharmacists
Source: BMC Cancer. 2017 Feb 10;17:122. doi: 10.1186/s12885-017-3110-2 (PMC5303208; doi:10.1186/s12885-017-3110-2)
Supplement: Additional file 1: Table S1. — Internal validity of non-validated questionnaires. This table describes the internal validity (Cronbach’s alpha) of the 8 Usual Care Domains (Knowledge, Awareness, Social Influence, Self-efficacy, Intention Formation, Implementation, Adverse Events Management, Facilitation), the Usual care sum score, the PAMQs sum score, the SDM score and the BMQ Necessity and BMQ Concerns subscales for all professions (physicians, nurse practitioners, nurses and pharmacists) as well as for all healthcare providers together. (DOCX 18 kb) [file 12885_2017_3110_MOESM1_ESM.docx]

| **Additional file 1: Table S1**. Internal validity of non-validated questionnaires. | | | | | | |  |  |  |  |  |
| --- | --- | --- | --- | --- | --- | --- | --- | --- | --- | --- | --- |
|  |  | physicians | | nurse practitioners | | nurses | | pharmacists | | All HCP | |
|  |  | n | Cronbach's  alpha | n | Cronbach's  alpha | n | Cronbach's  alpha | n | Cronbach's  alpha | n | Cronbach's  alpha |
| **Usual care domains** | |  |  |  |  |  |  |  |  |  |  |
| Knowledge | | 87 | 0.509*** | 33 | 0.762* | 58 | 0.823 | 46 | 0.841* | 194 | 0.836 |
| Awareness | | 79 | 0.712 | 32 | 0.569 | 58 | 0.667 | 46 | 0.683 | 195 | 0.693 |
| Social Influence | | 81 | 0.360 | 32 | 0.192 | 61 | 0.446 | 46 | 0.102 | 195 | 0.519 |
| Self-efficacy | | 85 | 0.886 | 32 | 0.868 | 61 | 0.926 | 46 | 0.821 | 195 | 0.886 |
| Intention Formation | | 84 | 0.699 | 31 | 0.666 | 61 | 0.732 | 46 | 0.805 | 190 | 0.754 |
| Implementation | | 78 | 0.840 | 32 | 0.468 | 61 | 0.571 | 45 | 0.679 | 193 | 0.548 |
| Adv Events Management | | 98 | 0.301** | 33 | 0.800* | 65 | 0.898 | 46 | 0.896 | 194 | 0.909 |
| Facilitation | | 77 | 0.698* | 32 | 0.680 | 57 | 0.864 | 46 | 0.806 | 191 | 0.791 |
| **Usual care sum score** | | 61 | 0.918**** | 29 | 0.909*** | 54 | 0.951 | 45 | 0.960* | 180 | 0.955 |
| **PAMQs sum score** | | 66 | 0.517 | 35 | 0.587 | 60 | 0.546 | 47 | 0.555 | 208 | 0.582 |
| **SDM score** | | 66 | 0.861 |  | n.a. |  | n.a. |  | n.a. |  | n.a. |
| **BMQ Necessity** | | 66 | 0.717 | 35 | 0.801 | 59 | 0.773 | 47 | 0.823 | 207 | 0.784 |
| **BMQ Concerns** | | 66 | 0.579 | 34 | 0.337 | 59 | 0.469 | 47 | 0.413 | 206 | 0.480 |
| Abbreviations: HCP, healthcare providers; PAMQs, Perceptions of Adherence Management Questions; SDM-score, sum | | | | | | | | | | | |
| score of the Shared Decision Making-doc-Questionnaire; BMQ, Beliefs about Medicines Questionnaire. | | | | | | | | | | |  |
| *. **, ***, ****; 1, 2, 3, 7 items were excluded from the analyses because the components had zero variance. | | | | | | | | | | |  |
